# Comparison of Intense Pulsed Light Therapy on Patients with Meibomian Gland Dysfunction Using AQUA CEL and M22 Devices

**DOI:** 10.3390/jcm11154265

**Published:** 2022-07-22

**Authors:** Shima Fukuoka, Reiko Arita

**Affiliations:** 1 Lid and Meibomian Gland Working Group (LIME), 626-11 Minami-Nakano, Minumaku, Saitama 337-0042, Japan; fshima3271@gmail.com; 2 Omiya Hamada Eye Clinic, 1-169-1 Sakuragicho, Omiyaku, Saitama 330-0854, Japan; 3 Department of Ophthalmology, The University of Tokyo, 7-3-1 Hongo, Bunkyoku, Tokyo 113-8655, Japan

**Keywords:** intense pulsed light, treatment, meibomian gland dysfunction, dry eye disease, meibomian gland expression

## Abstract

The purpose of this study was to compare the efficacy of Intense Pulsed Light (IPL) therapy for meibomian gland dysfunction (MGD) using the new AQUA CEL (AC, Jeisys) device and the traditional M22 (Lumenis) device. A total of 59 eyes of 59 patients with MGD (12 men and 47 women, mean age 49 ± 12 years) were enrolled. They randomly received four sessions of IPL therapy every three weeks either with AC (30 eyes) or M22 (29 eyes). Standard Patient Evaluation of Eye Dryness (SPEED) questionnaire score, noninvasive breakup time (NIBUT), lid margin abnormalities, corneal and conjunctival fluorescein staining, fluorescein breakup time (FBUT), Schirmer’s test, meiboscore and meibum grade were evaluated before treatment and one month after treatment. Before IPL, no significant differences were seen in age, gender, or measured parameters between the AC and M22 groups (*p* > 0.05, respectively). SPEED score, NIBUT, lid margin abnormalities, fluorescein staining, FBUT, and meibum grade improved significantly in both groups after IPL compared to before IPL (*p* < 0.001, respectively). There were no significant differences in measured parameters between the two groups after IPL (*p* > 0.05, respectively). IPL therapy with AC and M22 devices has been shown to be equally effective for the treatment of MGD.

## 1. Introduction

The International Workshop on Meibomian Gland Dysfunction defined meibomian gland dysfunction (MGD) as a chronic, diffuse abnormality of the meibomian glands that is commonly characterized by terminal duct obstruction or qualitative or quantitative changes in glandular secretion, which can result in changes to the tear film, clinically apparent inflammation, ocular surface disease, and symptoms of eye irritation [1]. Meibomian glands secrete the tear film lipid layer. Obstructive MGD is a major cause of lipid layer deficiency and evaporative dry eye (EDE) [2,3].

Intense pulsed light (IPL) therapy has been used in dermatology, especially in the cosmetic industry, to treat various skin conditions. IPL has been shown to improve subjective symptoms, stability of the tear film, inflammation of the eyelids, and meibomian gland secretion in patients with MGD and dry eye [4,5,6,7,8,9,10,11,12,13,14,15]. The TFOS DEWS II Management and Therapy Report recommended IPL as a second step therapy after education, lid hygiene, and different types of ocular lubricants [16]. The exact mechanisms underlying effects of IPL therapy on subjective symptoms and objective signs of MGD are still not well understood. It has been suggested that IPL attenuates telangiectasia, reduces Demodex, warms and melts meibum, modulates the concentrations of various pro- and anti-inflammatory factors, and suppresses matrix metalloproteinases (MMPs) [15]. There are several IPL devices commercially available for treating MGD. The M22 (Lumenis, Yokneam, Israel) and the E > Eye (E-SWIN, Houdan, France) are the two main devices. Several previous randomized control studies reported that IPL therapy using an M22 [5,7,10,11,17] or E > Eye [9,12,13,17] significantly improved subjective symptoms and objective signs in patients with MGD. Only one randomized study compared the effect of IPL therapy on patients with MGD, between M22 and E > Eye devices [17]. AQUA CEL (AC; Jeisys Medical, Seoul, Korea) has been commercially available in Korea since 2017 and in Japan since 2021. No paper has yet shown the efficacy of IPL therapy using AC on patients with MGD. The present study aimed to examine and compare the effect of AC and M22 devices for treating MGD.

## 2. Materials and Methods

### 2.1. Study Design

This retrospective randomized study was conducted at Itoh Clinic in Saitama, Japan, adhered to the tenets of the Declaration of Helsinki, and was approved by the Institutional Review Board of the Faculty of Medicine at Itoh Clinic (approval code: IRIN-202103). Written informed consent was obtained from all participants.

### 2.2. Subjects

Patients with MGD who attended Itoh Clinic between June and September 2021 were eligible for enrollment. The patients were consecutively enrolled in the study, with their baseline characteristics being found not to differ significantly among the treatment groups. Inclusion criteria were as follows: (1) at least 20 years of age; (2) diagnosis of MGD according to Japanese MGD diagnostic criteria [18] including ocular symptoms, plugged gland orifices, vascularity of lid margins, irregularity of lid margins, and decreased meibum quality and quantity (Shimazaki grading: meibum grade of ≥1, where grade 0 = clear meibum easily expressed, grade 1 = cloudy meibum expressed with mild pressure, grade 2 = cloudy meibum expressed with more than moderate pressure, and grade 3 = meibum could not be expressed even with strong pressure) [19]; (3) Fitzpatrick skin type of I to IV according to sun sensitivity and appearance of the skin [20]. Exclusion criteria included the presence of active skin lesions, skin cancer, or other specific skin pathology, active ocular infection or ocular inflammatory disease.

### 2.3. Experimental Design

MGD patients were randomly assigned to receive either IPL therapy with the new AC device or with the traditional M22 device as a control. Each patient underwent a series of four treatment sessions at three-week intervals. Each patient was subjected to clinical examinations as described below, both before treatment and one month after the four treatment sessions. All patients were asked to continue their current ocular medications. All patients used a warm compress and practiced lid hygiene at home twice a day during the study including the follow-up period. No patient was allowed to initiate therapy with a new topical or systemic agent for dry eye or MGD, other than a warm compress or lid hygiene, during the treatment course.

### 2.4. Clinical Assessment

For evaluation of treatment efficacy, the following parameters were measured sequentially before the first treatment and one month after the final treatment: (1) Symptoms were assessed with the Standard Patient Evaluation of Eye Dryness (SPEED) validated questionnaire (0–28) [21]. (2) Noninvasive breakup time (NIBUT) was determined with a DR-1α tear interferometer (Kowa, Nagoya, Japan), as described previously [22]. (3) Lid margin abnormalities (plugging of meibomian gland orifices and vascularity of lid margins) were observed with a slit-lamp microscope and were scored as previously described [23]. (4) The fluorescein-based breakup time of the tear film (FBUT) was measured after instillation of 1 μL of a preservative-free solution of 1% fluorescein dye into the conjunctival sac with the use of a micropipette, and the participants were asked to blink several times. FBUT was measured three times consecutively with a stopwatch, and the mean of the three values was calculated. (5) The corneal and conjunctival staining score (fluo score, 0–9) [24] based on fluorescein staining, and (6) meibum grade (0–3) [19] were evaluated with a slit-lamp microscope. (7) Morphological changes of the meibomian glands were assessed on the basis of the meiboscore for both eyelids (total of 0–6) [25] as determined by noninvasive meibography (DC-4, Topcon, Tokyo, Japan). (8) The volume of tear fluid was measured by Schirmer’s test, performed without anesthesia [26]. Only the data of the left eye of each subject were used. Eyes were categorized as showing an improvement (that is, treatment was effective) if the SPEED score had decreased by ≥4 points [27] and meibum grade had decreased by ≥1 point after treatment compared with before treatment.

### 2.5. IPL Procedure with M22 and AQUA CEL Devices

Before the first treatment, each patient underwent Fitzpatrick skin typing [20]. The IPL therapy was performed with an M22 or AC device, utilizing a 590 nm filter and a 15 × 8 mm lightguide for the upper eyelids (SapphireCool^TM^ tip for M22, Treatment Long Type for AC). The fluence of the IPL was adjusted respective to the appropriate setting of around 10 J/cm^2^ across the upper eyelids and 15 J/cm^2^ across the lower eyelids for M22, and around 15 and 20 J/cm^2^ for AC, respectively (Table 1). At each treatment session, both eyes of the patient were closed and sealed with disposable eye shields (AQUA CEL hydrogel eye care patch, KBM, Seoul, Korea). After generous application of ultrasonic gel to the targeted skin area, each patient received ~10 pulses of light (with slightly overlapping applications) from the right preauricular area, across the cheeks and nose, to the left preauricular area, reaching up to the inferior boundary of the eye shields. This procedure was repeated in a second pass. Each patient then received two passes of 3 pulses of light across the upper eyelids. Immediately after the IPL treatment, meibomian gland expression (MGX) was performed on both upper and lower eyelids of each eye with an Arita Meibomian Gland Expressor (Inami, Tokyo, Japan). Pain was minimized during MGX with the application of 0.4% oxybuprocaine hydrochloride to each eye.

### 2.6. Statistical Analysis

Data were found to be non-normally distributed with the Shapiro-Wilk test (*p* < 0.05), and nonparametric testing was selected. Fisher’s exact test was used to compare baseline categorical variables between the AC and M22 groups. The Mann-Whitney *U* test was used to compare pretreatment and posttreatment continuous variables between the AC and M22 groups. The Wilcoxon signed-rank test was used to compare variables before and after treatment. The outcome variables of the study were the SPEED score and the meibum grade before and after treatment. We performed a statistical power analysis for both the SPEED score and the meibum grade. For the SPEED score, the mean difference between the scores before and after treatment was 9.9, with a corresponding standard deviation (SD) of 1.5; for meibum grade, the mean difference was 1.9 with an SD of 0.6. These changes were calculated from the results of all 59 eyes in the current study. The number of eyes in each group for the power analysis was assumed as 29. The power (1 − β) was 1.0 at the level of α = 0.05 for both the SPEED score and the meibum grade, and the sample size was sufficient. Statistical analysis was performed with JMP Pro version 16 software (SAS, Cary, NC, USA). Data are shown as means ± SDs. All statistical tests were two sided, and a *p* value of < 0.05 was considered statistically significant.

## 3. Results

### 3.1. Baseline Characteristics

Patient baseline characteristics are shown in Table 2. Fifty-nine eyes of 59 MGD patients, including 47 women and 12 men, were enrolled in the study. The mean age ± SD was 49.2 ± 11.8 years (range of 23–71 years). The mean duration of MGD ± SD was 3.8 ± 1.5 years (range of 1–8 years). Participants randomly received four sessions of IPL and MGX every three weeks with either AC (30 eyes) or M22 (29 eyes) devices. None of the characteristics at baseline differed significantly between the two groups (Table 2). Approximately 60% of patients had a history of ocular surgery such as cataract removal or LASIK (Table 2). The frequency of other MGD and dry eye therapies previously administered is shown in Table 3, with most patients having been treated with a warm compress, lid hygiene, topical steroids, diquafosol eyedrops, or rebamipide eyedrops.

### 3.2. Efficacy of IPL with M22 and AQUA CEL

The characteristics of the eyes in the AC group and the M22 group before as well as one month after the final treatment session are shown in Table 4. No significant differences in parameters were detected between the two groups before treatment. The SPEED score was significantly reduced after treatment compared with pretreatment in both groups (*p* < 0.001, respectively) but did not differ significantly between the two groups after treatment (*p* = 0.052). Significant increases in NIBUT and FBUT as well as significant decreases in plugging, vascularity, fluo score, and meibum grade were also apparent after treatment in both groups (*p* < 0.001, respectively). These parameters did not differ significantly between the two groups after treatment (*p* = 0.70, 0.31, 0.052, 0.51, 0.32, 0.44, respectively). Meiboscore was not significantly improved after treatment compared with pretreatment in both groups (*p* = 0.16, respectively), and did not differ significantly between the two groups after treatment (*p* = 1.0). An improvement in Schirmer’s test value was observed after the treatment session only in the AC group (*p* = 0.005), with such an improvement not being observed in the M22 group (*p* = 0.16). An improvement in Schirmer’s test value after treatment did not differ significantly between the two groups (*p* = 0.23).

### 3.3. Adverse Events

There were no adverse events related to the device or the procedure in both groups.

## 4. Discussion

This is the first study to show that IPL therapy with an AC device significantly improved subjective symptoms, lid margin abnormalities, tear film stability, corneal and conjunctival epithelial damage, and meibomian gland function. There is no difference in safety and efficacy between the AC and the M22.

Prospective randomized controlled studies reported the efficacy of IPL treatment on MGD using M22 [5,7,11,17] and E > Eye [9,12,13,17] devices. Only one randomized study reported that IPL treatment with an M22 was more effective in improving meibomian gland function in lower eyelids and tear film stability than that with an E > Eye [17]. In other studies, patients in the control group received sham IPL treatment with [5,10,11] or without [9,12,13] MGX, or MGX only [7]. There are two IPL devices available for ophthalmological use in Japan. One is an M22 and the other is an AC. The AC has been commercially available in Korea since 2017 and in Japan since 2021. In Japan, the M22 was approved in the field of dermatology in 2013 by the Pharmaceuticals and Medical Devices Agency and has been used for ophthalmological use since 2016. The AC was approved in 2021. In this study, we performed IPL therapy according to Dr. Toyos’s setting [6] for the M22 and the manufacturers’ instructions for the AC. Most of the settings were the same except for the fluence (Table 1). There are several differences between the two IPL devices. We used the lightguide of the same size, 8 × 15 mm, for the AC and the M22 (Table 1). There are different spot sizes of lightguides for the two IPL devices. M22 has a rectangular 15 × 35 mm and 6 mm cylindrical lightguide and AC has a 10 × 40 mm lightguide. The small lightguide for the AC is an attachment-type lightguide and longer than that for the M22. When using a different size lightguide, the lightguide at the tip of the handpiece is removed and replaced in the M22. By using a lightguide with a small contact area, it is possible to irradiate safely while avoiding eyebrows and eyelashes, even when treating the upper eyelid. On the other hand, a smaller lightguide is attached over the lightguide at the tip of the handpiece in the AC. Due to this distance with the small attachment-type lightguide, the AC has more energy loss. The fluence of IPL with a small lightguide for the AC is recommended to be higher than that for the M22. The M22 system features Optimal Pulse Technology (OPT^TM^) using a modular laser multi-application platform that ensures constant and reproducible fluence throughout the entire pulse [17]. A handpiece thermokinetic cooling system of the sapphire lightguide can be employed to provide epidermal protection, while at the same time allowing greater fluences to reach deeper targets for both devices. The M22 has a continuous contact cooling system. The AC has an Automatic Temperature Controller (ATC^TM^) that maintains skin surface temperature equally by controlling the sensor, which is monitored in real time.

In our study, we gave each patient a series of four IPL treatment sessions at three-week intervals. Although the standard optimal IPL protocol for MGD has not yet been established, we have created a protocol based on the previous review paper [14] as a widely recognized protocol at this stage.

We found that subjective symptoms improved significantly in both the AC and M22 groups after IPL, compared to before IPL. There was no significant difference in subjective symptoms between the two groups after IPL. Three prospective randomized controlled studies reported that IPL therapy improved subjective symptoms to a significantly greater extent compared with MGX alone [7] or with sham IPL treatment without MGX [12,13]. Three prospective randomized controlled studies reported that SPEED scores improved after treatment compared to the baseline in both IPL group and control groups, but changes in SPEED scores were similar in both groups [5,9,11].

We found that vascularity of the lid margin improved significantly after IPL with AC and M22, compared to before IPL. There were no significant differences in vascularity between the two groups after IPL. In our previous study, we found that IPL–MGX improved vascularity, whereas MGX alone did not [7]. The efficacy of IPL–MGX is suspected to be due to the anti-inflammatory effect of IPL [10,15].

Tear film stability is assessed by FBUT and NIBUT. We found that NIBUT and FBUT significantly improved after IPL with AC and M22, compared to before IPL. There were no significant differences in these parameters between the two groups before and after IPL. MGX alone improved tear film stability [7]. Three prospective randomized controlled studies reported that the effects of IPL with MGX treatment on FBUT was significantly greater than those of MGX alone [7] and those of sham IPL with MGX treatment [5,11]. It has been reported that IPL therapy with [7] or without [9] MGX improved NIBUT compared to control. FBUT and NIBUT was shown to be shorter in eyes with MGD than in healthy eyes. In our previous studies, we have reported that IPL improved the tear film lipid layer thickness and the tear interferometric pattern, indicating that IPL recovered the balance in tear film components between the lipid and aqueous layers of the tear film [7,8]. We believe that this improvement in tear film homeostasis led to an improvement in tear film stability.

We found that the fluorescein staining score significantly improved after IPL with AC and M22 compared to before IPL. There was no significant difference in the fluorescein staining score between the two groups before and after IPL. One prospective randomized controlled study reported that corneal and conjunctival fluorescein staining scores improved significantly after treatment in the IPL with MGX group, but not after MGX alone [7]. Two prospective randomized controlled studies reported that corneal fluorescein staining scores improved after treatment compared to the baseline in both the IPL group and the sham group, but corneal fluorescein staining scores did not differ between the two groups [5,11]. Another prospective randomized controlled study reported that no significant improvement was seen in corneal and conjunctival epithelial damage after IPL [12]. MMPs have important roles in corneal wound healing. IPL is thought to downregulate tumor necrosis factor (TNF)-α and indirectly diminish the levels of MMPs [14].

We found that plugging and meibum grade improved significantly after IPL with AC and M22, compared to before IPL. There were no significant differences in these parameters between the two groups before and after IPL. In our previous study, we reported that plugging and meibum grade significantly improved after both IPL with MGX and MGX only [7]. A significantly better improvement in these parameters was seen in the IPL with MGX group compared to the control group [7]. Two previous studies reported that IPL significantly improved meibomian gland yielding secretion score compared to baseline [5,11]. One study reported that IPL significantly improved meibum quality and meibomian gland expressibility [13]. The broad wavelength of IPL can be absorbed by melanin and hemoglobin in human skin, to develop heat [15]. The temperature elevation of eyelid skin and meibomian glands could melt meibum and make it easier to be secreted [14].

In our study, meiboscore did not show significant improvement after treatment compared to before IPL in either group. There were no significant differences in meiboscore between the two groups before and after IPL. In two previous studies, meiboscore remained unchanged after IPL [5,12]. Our previous study reported that meiboscore improved significantly only in the IPL group, but there was no significant difference in meiboscore between the IPL group and the MGX only group [7]. Another previous study reported that meiboscore significantly improved in both the IPL group and the sham IPL group, but meibomian grades in the two groups were not statistically different at each timepoint [13]. Further research is needed to determine whether IPL could improve meibomian gland morphology by reducing the risk of physical obstruction of the meibomian glands.

In our previous study, we reported that the Schirmer’s test value did not change after IPL compared to baseline and did not differ significantly between the IPL and control groups [7]. In this study, the Schirmer’s test value improved after the IPL treatment session only in the AC group. There was no significant difference in the Schirmer’s test value between the two groups before and after IPL. The change in the Schirmer’s test value was less than 1 mm. It was statistically significant but may be unimportant from a clinical standpoint. Two previous studies reported that there was no difference in tear meniscus height from baseline in either the IPL or the sham treated groups [9,12].

In our study, there have been no complications such as burns. IPL therapy has been mainly applied to the lower eyelids to avoid the possibility of damaging intraocular tissues by broad-spectrum light [15]. Recently, several studies have reported the safety of IPL application on the upper eyelids [4,5,7,10]. Mild transient pain and skin redness as well as partial eyelash loss was reported after IPL treatment to both upper and lower eyelids, with a larger lightguide and a lid plate [5]. In our study, we were able to safely apply IPL directly on the upper and lower eyelids using a smaller lightguide and disposable eye shields, as previously reported [6].

Several limitations of this study should be acknowledged. First, our study was retrospective. Second, the mechanisms underlying the improvement in subjective and objective parameters of the two IPL devices were not elucidated in this study. Third, the follow-up period was relatively short. In addition, our patients were only Japanese. Most Japanese people are classified as Fitzpatrick skin type III. The reactivity of the skin to light or to ultraviolet light may differ between the study patients and individuals of other ethnicities. We did not compare various protocols for the best treatment efficacy with each IPL device. Further investigation is required to validate these findings. Larger prospective randomized clinical studies with a longer follow-up period will be needed to compare the efficacy of the IPL devices and to develop standard treatment protocols for the use of IPL therapy.

## 5. Conclusions

Patients with MGD showed improvement in both subjective symptoms and objective signs of MGD after IPL therapy with AC and M22 devices followed by MGX. Our results suggest IPL therapy with AC and M22 devices has been shown to be equally effective for the treatment of MGD.

## Figures and Tables

**Table 1:** Differences in systems and settings between the two intense pulsed light (IPL) devices.

Descriptions	M22	AQUA CEL
Pulse technology	Optimal Pulse Technology (OPT^TM^)	
Wavelength filter (nm)	590	590
Spot size (mm)	8 × 15	8 × 15
Lightguide	Sapphire crystal tip (SapphireCool^TM^)	Sapphire crystal tip (Treatment Long Type)
Fluence for the upper eyelids (J/cm^2^)	10	15
Fluence for the lower eyelids (J/cm^2^)	15	20
Number of pulses	Triple	Triple
Pulse width (ms)	6.0/6.0/6.0	6.0/6.0/6.0
Delay time (ms)	50	50
Cooling system	Continuous contact cooling	Automatic Temperature Controller (ATC^TM^)

**Table 2:** Baseline characteristics of the intense pulsed light (IPL) therapy with M22 and AQUA CEL groups of study subjects with meibomian gland dysfunction (MGD).

	All (*n* = 59)	M22 Group (*n* = 29)	AQUA CEL Group (*n* = 30)	*p* Value
Age, mean ± SD (range) (years)	49.2 ± 11.8 (23–71)	49.2 ± 11.7 (32–71)	49.2 ± 12.1 (23–67)	0.70
Sex (male/female)	12/47	6/23	6/24	1.0
Duration of MGD, mean ± SD (range) (years)	3.8 ± 1.5 (1–8)	3.8 ± 1.4 (2–8)	3.9 ± 1.6 (1–7)	0.64
Previous ocular surgery (eyes (%))	34 (57.6%)	17 (58.6%)	17 (56.7%)	1.0

*p* values were obtained with Mann-Whitney *U* test or Fisher’s exact test. SD, standard deviation.

**Table 3:** Previous therapies for the study patients in M22 and AQUA CEL groups.

Therapy	M22 Group (*n* = 29)	AQUA CEL Group (*n* = 30)	*p* Value
Warm compress	28 (96.6%)	29 (96.7%)	1.0
Lid hygiene	15 (51.7%)	18 (60%)	0.60
Meibomian gland expression	2 (6.9%)	2 (6.7%)	1.0
Fluoromethorone eyedrops	29 (100%)	30 (100%)	1.0
Diquafosol eyedrops	20 (69.0%)	20 (66.7%)	1.0
Rebamipide eyedrops	16 (55.2%)	17 (56.7%)	1.0
Preservative-free artificial tears	13 (44.8%)	11 (36.7%)	0.60
Levofloxacin eyedrops	11 (37.9%)	10 (33.3%)	0.79
Olopatadine eyedrops	6 (20.7%)	5 (16.7%)	0.75
Hyaluronic acid eyedrops	3 (10.3%)	3 (10.0%)	1.0
Ofloxacin ophthalmic ointment	2 (6.9%)	2 (6.7%)	1.0
N-3 fatty acid supplementation	13 (44.8%)	9 (30.0%)	0.29

*p* values were obtained with Fisher’s exact test.

**Table 4:** Comparison of M22 and AQUA CEL groups before and one month after the final treatment session.

		Pretreatment		After Treatment			
Characteristic	Group	Mean ± SD	*p* Value for M22 vs. AQUA CEL	Mean ± SD	Mean Change± SE	*p* Value vs. Pretreatment	*p* Value for M22 vs. AQUA CEL
SPEED score	M22	13.8 ± 3.3	0.75	4.3 ± 1.6	−9.5 ± 0.7	<0.001 **	0.052
(0–28)	AQUA CEL	13.9 ± 2.4		3.6 ± 1.3	−10.3 ± 0.4	<0.001 **	
Plugging	M22	2.1 ± 0.9	0.72	0.5 ± 0.5	−1.6 ± 0.1	<0.001 **	0.052
(0–3)	AQUA CEL	2.1 ± 0.9		0.3 ± 0.4	−1.9 ± 0.2	<0.001 **	
Vascularity	M22	1.6 ± 0.8	0.95	0.4 ± 0.5	−1.2 ± 0.1	<0.001 *	0.51
(0–3)	AQUA CEL	1.6 ± 0.7		0.5 ± 0.5	−1.1 ± 0.1	<0.001 **	
NIBUT	M22	2.6 ± 1.3	0.58	5.6 ± 2.6	3.0 ± 0.5	<0.001 **	0.70
(s)	AQUA CEL	2.7 ± 1.0		5.9 ± 1.8	3.1 ± 0.4	<0.001 **	
FBUT	M22	3.7 ± 1.2	0.62	6.8 ± 1.6	3.1 ± 0.3	<0.001 **	0.31
(s)	AQUA CEL	3.5 ± 1.3		6.4 ± 1.5	2.9 ± 0.4	<0.001 **	
Fluo score	M22	1.9 ± 1.6	0.76	0.4 ± 0.8	−1.5 ± 0.2	<0.001 **	0.32
(0–9)	AQUA CEL	1.9 ± 2.1		0.3 ± 0.8	−1.7 ± 0.3	<0.001 **	
Meiboscore	M22	4.3 ± 1.3	0.99	4.3 ± 1.3	−0.1 ± 0.0	0.16	1.0
(0–6)	AQUA CEL	4.3 ± 1.3		4.3 ± 1.3	−0.1 ± 0.0	0.16	
Meibum grade	M22	2.4 ± 0.7	0.86	0.4 ± 0.7	−1.9 ± 0.1	<0.001 **	0.44
(0–3)	AQUA CEL	2.4 ± 0.8		0.5 ± 0.5	−1.9 ± 0.1	<0.001 **	
Schirmer’s test value	M22	7.1 ± 6.7	0.53	6.3 ± 5.0	−0.8 ± 0.5	0.16	0.23
(mm)	AQUA CEL	7.1 ± 5.1		8.0 ± 5.8	0.9 ± 0.3	0.005 *	

*p* values were determined with the Mann-Whitney *U* test or the Wilcoxon signed-rank test. * *p* < 0.05, ** *p* < 0.001. SD, standard deviation; SE, standard error; SPEED, Standardized Patient Evaluation of Eye Dryness; NIBUT, noninvasive breakup time of the tear film; FBUT, fluorescein-based breakup time of the tear film; fluo score, corneal-conjunctival fluorescein staining score.

## Data Availability

The datasets generated during and analyzed in the current study are available from the corresponding author on request.
